# Precision Detection Technology: Equipping Precision Oncology with Wings

**DOI:** 10.1155/2020/9068121

**Published:** 2020-07-09

**Authors:** Rilan Bai, Zheng Lv, Xiao Chen, Hanfei Guo, Ling Bai, Huimin Tian, Wei Li, Jiuwei Cui

**Affiliations:** Cancer Center, The First Hospital of Jilin University, Changchun, Jilin 130021, China

## Abstract

In recent years, precision medical detection techniques experienced a rapid transformation from low-throughput to high-throughput genomic sequencing, from multicell promiscuous detection to single-cell precision sequencing. The emergence of liquid biopsy technology has compensated for the many limitations of tissue biopsy, leading to a tremendous transformation in precision detection. Precision detection techniques contribute to monitoring disease development more closely, evaluating therapeutic effects more scientifically, and developing new targets and new drugs. In the future, the role of precision detection and the joint detection in epigenetics, rare gene detection, individualized targeted therapy, and multigene targeted drug combination therapy should be extensively explored. This article reviews the changes in precision medical detection technology in the era of precision medicine, as well as the development, clinical application, and future challenges of liquid biopsy.

## 1. Introduction

Precision medicine development should be based on accurate detection and should take into account various factors, including genetic and environmental susceptibly factors including living habits in order to identify disease etiology, pathogenesis, and therapeutic targets. The aim of precision medicine is to eventually improve the benefits of disease in diagnosis, treatment, and prevention, as well as achieve optimal personalized precision treatment [1]. The era of precision medicine has brought tremendous changes for the diagnosis and treatment of cancer. The discovery of tumor-driven genes has led to the development of targeted therapeutic drugs that have promoted individualized precision treatment [2] but have also brought challenges to precision medical detection technology. In recent years, clinical demands have gradually driven the development and application of precision detection techniques and promoted the emergence of new technologies, new targets, and new drugs in these times of precision medicine.

## 2. The Development of Precision Sequencing Technology

Given that different pathological tumor types have different gene mutations, precision medical detection technology has been revolutionized from the detection of mutations at a single site to multiple sites simultaneously, from low-throughput to high-throughput sequencing and from multicell hybrid detection to single-cell precision detection. It has achieved the simultaneous detection of multitarget detection of genes, which promotes the development of precision medicine.

### 2.1. Low-Throughput Genomic Sequencing to High-Throughput Genomic Sequencing

The first-generation sequencing technology (Sanger sequencing) was difficult to universalize as it was expensive and time consuming. The constant demand of the clinic led to the emergence of next-generation sequencing (NGS) that can obtain all the genetic information and provide a “real-time” snapshot of the tumor genetic landscape by combining polymerase chain reaction (PCR) with fluorescent labeling imaging technology [3]. Currently, many NGS-based tumor gene panels have been used in clinical practice, such as FoundationOne CDx detecting 324 gene mutations and MSK-IMPACT detecting 468 gene mutations approved by the U.S. Food and Drug Administration (FDA). Although there are many advantages, NGS is technically limited because the reading length is short, which would cause regional assembly errors of high complexity of the genome, increased sequencing gap area, and difficulty in detection of low abundance. Not only that, NGS would introduce many potential deviations and errors during data reading and sequence assembly due to the need of amplification and segmentation during the process of database construction [4].

The third-generation single-molecule sequencing technology has gradually developed [5]. It compensates for the disadvantage of shorter read length of the second-generation sequencing and does not require PCR amplification, allowing rapid sequencing splicing of whole genome sequences (WGS), avoiding the above sequencing deviations and being more advantageous in sequencing cost and time. This technology can also obtain both genetic and epigenetic information at the single nucleotide level from DNA, providing important technical support for the in-depth analysis of the epigenetic mechanisms of different metabolic processes in organisms. Nevertheless, the analysis of epigenetic information at the single nucleotide level is not the exclusive characteristic of single-molecule sequencing. In tumor detection, single-molecule sequencing technology can detect not only genetic mutations but also epigenetic modification sites and the presence of long-chain noncoding RNAs, allowing tumor classification and grading [6, 7]. RNA-sequencing (RNA-seq) is a relatively new application of NGS that is gradually replacing microarrays as the preferred technology to analyze transcripts. RNA-seq can quantify gene expression, detect new transcripts, and analyze small noncoding RNAs that are not translated into proteins [8, 9]. Large RNA-seq datasets using whole-blood RNA-seq combined with variant and phenotypic-related genes have practical value in identifying abnormal expression, splicing, and alternative splicing events in rare disease candidate genes [10].

### 2.2. Multicell Hybrid Detection to Single-Cell Precision Detection

Traditional genetic testing is based on mixed-cell DNA samples. The results of this analysis represent the characteristics of most cells in the tissue or the average of the whole sample, making it impossible to characterize the intercellular differences and interpret the growth and development mechanism of the organism and the features of a small number of special cells. In recent years, single-cell sequencing technology, based on high-throughput sequencing analysis of the genome, transcriptome, and epigenetics of single cells, has allowed to analyze tumor heterogeneity and clonal evolution and to reveal the molecular mechanisms of tumor chemotherapy resistance [11]. Single-cell sequencing technology can detect microgenes and rare noncoding RNAs and can reveal the evolution of individual cells at specific times that helps to understand the occurrence and evolution of tissues or organisms at the individual level [11–13].

Single-cell sequencing allows the construction of immune cell maps and the study of the tumor immune microenvironment [14–16]. Guo et al. [15] used single-cell sequencing to study the subpopulation, baseline of lineage, and functional status of tumor infiltrating lymphocytes (TILs) and designed the non-small cell lung cancer (NSCLC) T-lymphocyte map. It includes a single-cell data acquisition process and immune profiles such as T-cell subset classification, state transitions between subpopulations, and heterogeneity of regulatory T-cells. In addition, single-cell sequencing can also reveal cellular spatial localization. Keren et al. [17] obtained single-cell level information by isotope labeled proteins, named multiplexed ion beam imaging by time of flight (MIBI-TOF). This technology allows to systematically understand the spatial distribution characteristics of breast cancer cells and different types of immune cells to accurately visualize the cell distribution characteristics of different patients and to further evaluate the potential efficacy of immunotherapy and the prognosis of a disease.

## 3. Revolutionary Changes Brought by Liquid Biopsy Technology for Precision Medical Testing

Due to the continuity of spatiotemporal and dynamic evolution, tumor cells and genomic variation appear highly heterogeneous. Tumor heterogeneity is broadly classified into inter- and intratumor heterogeneity [18, 19]. The high heterogeneity and rapid evolution of tumors is the biggest obstacle to accurate detection and diagnosis of tumors [20, 21]. Sampling area deviation may occur during tissue biopsy, that is, one regional tumor feature cannot represent the entire tumor, and continuous extensive tissue sampling of primary and metastatic lesions is difficult to achieve. In addition, tissue biopsy is limited to the characteristics of invasive and static detection, so it is urgent to find a more comprehensive testing method that reflects the characteristics and dynamic changes of tumors.

In recent years, the emergence of liquid biopsy technology seems to enable the precision detection from static to dynamic. It allows to repeatedly and dynamically monitor the evolution of tumors over time and become the most powerful tool for studying intratumor heterogeneity by simple noninvasive blood sampling and analysis [22, 23]. This technology uses PCR or high-throughput sequencing to detect circulating tumor DNA (ctDNA), circulating tumor cells (CTCs), extracellular vesicles (EVs), and circulating tumor RNA (ctRNA) released from the primary tumor and metastatic sites into the bloodstream [24, 25]. Clinically, liquid biopsy can achieve comprehensive and dynamic monitoring of tumor markers, so it has significant clinical applications in tumor screening and early detection, real-time monitoring of tumor treatment, researching new treatment targets and drug resistance mechanisms, and predicting risks of metastatic recurrence. Although some studies have confirmed the potential of liquid biopsy to change the mode of cancer treatment, future investigations are needed to fully evaluate its role in tumor detection and treatment [26].

### 3.1. The Detection and Clinical Application of ctDNA

#### 3.1.1. The Development of ctDNA Detection Technology

Quantitative information can be obtained from the measurement of the mutant allele fraction (MAF), which is a reflection of the tumor burden. It is used to detect minimal residual disease (MRD) and occult metastases; it is also a high-sensitivity, high-specificity predictor in monitoring therapeutic effectiveness and relapse. Qualitative information can be sourced through the profiling of mutations, amplifications, deletions, and translocations in ctDNA. This allows the identification of genetic alterations associated with response, hence supporting decision making for personalized management [25]. In recent years, a variety of detection methods of ctDNA epidermal growth factor receptor (EGFR)-mutation have been developed, including the amplification refractory mutation system (ARMS), droplet digital PCR (ddPCR), and NGS [27]. ARMS is an economical and rapid method approved by the FDA for clinical testing, but its sensitivity is only about 50% [28–30]. ddPCR and NGS have significantly improved detection accuracy, but the operation process is too complicated and expensive and is therefore not widely used in clinical practice. The SuperARMS, an improved method of ARMS, was recently proposed with enhanced sensitivity and a simplified operation process. China FDA (CFDA) has approved it to detect EGFR mutation status in NSCLC patients to screen for those suitable for targeted drugs [31]. Li et al. calculated the clinical efficacy of the SuperARMS EGFR test using EGFR mutation status in tumor tissue as a standard reference. The results showed that EGFR mutations were detected in 45.9% (50/109) of plasma samples and 56.9% (62/109) of matched tumor tissue samples and verified the sensitivity (82%), specificity (100%), positive predictive value (100%), and negative predictive value (81.4%) of SuperARMS [31]. The AURA 17 expansion study (NCT01802632) used Cobas, SuperARMS, and ddPCR to detect ctDNA EGFR gene mutations in NSCLC cases of the Asia-Pacific population. The results showed the positive detection rates of the three blood tests compared to the tissue test results, which were 42%, 49%, and 56%, respectively. The objective response rate (ORR) of osimertinib in T790M-positive patients was 56%, 64%, and 56%, respectively. This study confirms the accuracy of SuperARMS in the prediction and guidance of clinical medication, proposing it as the optimal choice for clinical blood ctDNA testing.

In addition to single-gene detection, ctDNA-based multigene analysis techniques, such as blood TMB (bTMB) and microsatellite instability (MSI) tests, show more convenient, fast, and dynamic characteristics compared with histological detection [32]. Recent results from POPLAR and OAK clinical studies have shown that bTMB can predict the efficacy of immunotherapy and that patients are more likely to benefit from immunotherapy as the cutoff value of bTMB increases [33, 34].

#### 3.1.2. The Clinical Applications of ctDNA

*(1) Early Diagnosis of Tumor*. Early screening of tumors through screening tests is a key step in early diagnosis and treatment of tumor. Early screening tests should be highly sensitive, specific, and cost-effective. However, a concern in early detection is the predictive value of single or small sets of mutations, since cancer-associated mutations can also be found in the plasma of healthy individuals as a result of clonal hematopoiesis [30]. One approach to overcome this challenge is the CancerSEEK platform recently proposed by Cohen et al. [35], which detects eight common cancer types by detecting rare mutations and tumor-derived protein biomarkers expected to be present in plasma ctDNA. The assay platform evaluates levels of eight proteins and the presence of mutations in 1933 different genomic locations, using rigorous statistical methods to ensure the accuracy of the test, such as logistic regression algorithm, 10 iterations of 10-fold cross-validations. The results showed that a median sensitivity of CancerSEEK tests was 70% of the eight cancer types, ranging from 69% to 98% for the detection of five cancer types (ovary, liver, stomach, pancreas, and esophagus), and the specificity was more than 99%. However, this study contains some limitations, and the sensitivity and false positive rate of the test require further research to verify.

*(2) Guidance for Tumor Treatment and Prediction of Therapeutic Efficacy*. The phase II BENEFIT study [36] proposed that dynamic monitoring of ctDNA changes can predict tumor treatment efficacy. The results showed that progression-free survival (PFS) was significantly longer in patients whose peripheral blood ctDNA EGFR mutations disappeared after 8 weeks of receiving first-line gefitinib. In addition, dynamic monitoring of ctDNA in immunotherapy can help to follow tumor residual disease, identify pseudoprogression, and predict the efficacy of immunotherapy. Lee et al. [37] confirmed that the changes of the ctDNA spectrum after 12 weeks of treatment could accurately distinguish between pseudoprogression and true disease progression. Using ctDNA abundance to identify pseudoprogression showed 90% sensitivity and 100% specificity, while the lactate dehydrogenase (LDH) profile showed 60% sensitivity and 89% specificity. They also verified that there was a significant difference in 1-year survival rates between patients with benign and malignant ctDNA changes (82% and 39%) [37].

*(3) Prediction of Tumor Recurrence and Metastasis*. Coombes et al. [38] performed plasma ctDNA sequencing (ultradeep sequencing) on 49 patients with postoperative or adjuvant breast cancer every 6 months and monitored CT imaging, liver function tests (LFT), and CA15-3 levels. The results showed that the specificity of breast cancer recurrence by ctDNA was 100% and the sensitivity was 89%, which was more accurate than the other three traditional detection methods. Another study confirmed that the continuous monitoring of ctDNA levels using the Safe-SeqS technique during postoperative follow-up in patients with stage III nonmetastatic CRC could identify disease recurrence prior to routine imaging studies [39]. In this study cohort, all 8 patients with recurrence had positive ctDNA during follow-up (100%, 95% CI, 63%–100%), whereas 5 of 8 patients with recurrence had positive CEA levels (>5 *μ*g/L) (63%; 95% CI, 24%–91%). Another study used NGS to detect mutations in serum samples at 1 and 3 months from 53 patients with acute myeloid leukemia and myelodysplastic syndrome (AML/MDS) receiving allogeneic hematopoietic stem cell transplantation (alloSCT), and at least one dPCR was performed in each case. The results showed persistent mutations in the bone marrow at 1 and 3 months [mutation persistence (MP): MP1 and MP3], and matched serum [ctDNA persistence (CP): CP1 and CP3] after alloSCT was associated with a higher 3-year cumulative recurrence rate, confirming the effectiveness of ctDNA in predicting the risk of disease recurrence [40].

#### 3.1.3. Methylation Detection in ctDNA Broadens the Field of Precision Medical Research

DNA methylation is one of the earliest epigenetic changes observed in tumor development, and its abnormal pattern is highly specific in different tumor tissues [41, 42]. Previous studies of epigenomics mainly relied on microarray technology, but the advent of NGS has largely facilitated the study of high-throughput data to detect epigenetic changes across the genome at single nucleotide resolution. Detection techniques for gene methylation include PCR, whole-genome methylation sequencing, and 450K methylation chips. Clinically, ctDNA methylation can reflect the hypermethylation characteristics of the promoter region of a gene, and the dual signals of tumor markers and tissue-specific methylation patterns can be used to detect and localize tumors. Additionally, detection of ctDNA methylation can be used for early tumor diagnosis and assessment of prognosis, as it can reflect tumor load changes [43, 44]. The most widely used lung cancer-specific gene methylation detection kit is the human SHOX2 and RASSF1A gene methylation DNA detection kit [45], which could assist in the early diagnosis of lung cancer and in the differentiation between benign and malignant pulmonary nodules. Additionally, together with a cytological test, it can effectively improve the sensitivity and specificity of lung cancer detection. Studies have shown that the combined detection of multiple gene methylation markers can help to improve the sensitivity and specificity of lung cancer diagnosis, reaching 71.5%–83.2% and 90%–97.4%, respectively [46]. A large-scale clinical data analysis study proposed methylation models for early screening, risk assessment, and prognosis monitoring of liver cancer, including a comprehensive diagnostic model (cd-score) and a comprehensive prognostic model (cp-score), and verified its potential role in tumor monitoring [47].

### 3.2. The Detection and Clinical Application of CTCs

CTCs are defined as cells that shed from the primary tumor site or from metastases and enter the blood circulation through the vascular or lymphatic system [48]. Their presence is considered the basis for tumor metastasis development. CTCs undergo vascular leakage by epithelial-mesenchymal transition (EMT) [49] or passive shedding of primary tumors. The latter mechanism is supported by the presence of CTCs aggregates or circulating tumor microemboli (CTM) in the blood [50]. In clinical practice, CTCs can be isolated and enriched through their biological and physical properties such as size, density, and charge or through specific molecules on their surface based on antigen-antibody recognition. Once identified, their nucleic acid can be analyzed by cytochemical staining, fluorescence in situ hybridization, PCR, and sequencing, among others. At present, the only detection technology of CTCs approved by the FDA for clinical use is the Cell Search system [51], which defines CTCs as epithelial cell adhesion molecule (EpCAM)+/cytokeratin (CK)+/4,6-diamidino-2-phenylindole (DAPI)+/CD45+. CTCs can provide clinical quantitative information through cell counting and qualitative information based on single-cell genome, transcriptomics, and proteomic analysis.

CTCs can reflect the tumor tissue and can help carry out pathological diagnoses, molecular sequencing, and disease monitoring, as well as providing treatment decision making and prognosis information for the clinic through noninvasive dynamic monitoring [52]. Scher et al. [53] performed blood CTCs and immunofluorescence staining on 142 patients treated with ARS inhibitors or taxanes and detected the expression of androgen receptor splice variant 7 (AR-V7) in CTCs. The overall survival (OS) of AR-V7-positive patients treated with taxanes was higher than in those treated with an androgen receptor signaling (ARS) inhibitor (median OS 14.3 vs. 7.3 months, *p* = 0.25), while AR-V7-negative patients treated with ARS inhibitors had a higher OS than those treated with taxanes. It is suggested that future the research studies of CTCs should look into the analysis of macromolecules expressed by CTCs and the design of therapeutic measures to target them.

### 3.3. The Detection and Clinical Application of EVs

EVs are lipid vesicles secreted by cells that carry various proteins and nucleic acids, which can provide tumor-related information [54, 55]. Studies have shown that PD-L1riched exosomes secreted by tumor cells can enter the peripheral blood of the body and inhibit the T-cells outside of the tumor microenvironment. Therefore, detecting EVs could help understand the mechanism of tumor immune escape and consequently develop targeted therapies to overcome immunosuppression [56]. Liquid biopsy technology of exosomes, known as ExoDx Prostate IntelliScore (EPI), has been approved by the FDA for the detection and prognosis assessment of prostate cancer. The study also showed that the Friend leukemia virus integration 1 (FLI1) exon cyclic RNA is a new carcinogenic driver that promotes tumor metastasis via the miR584-ROCK1 pathway. The content of FECR1 in serum exosomes is related to the therapeutic efficacy and prognosis of patients with small cell lung cancer and could therefore be used as a new marker for efficacy monitoring and prognosis evaluation [57, 58].

### 3.4. Combined Detection of Liquid Biopsy Expands the Application of Precision Medicine

Genomics combined with transcriptomics or proteomic analysis extends the application of precision medicine to provide the best treatment options for cancer. The WINTHER trial [59] recommends precise treatments for refractory tumors by integrating DNA and RNA gene expression profiles. Transcriptomics significantly increased the proportion of patients receiving matched treatment, and 22.4% of patients that received the recommended treatment had a PFS ratio of previously treated >1.5. CancerSEEK, a blood test, can detect eight common cancer types through assessment of the levels of eight circulating proteins and sixteen mutations in ctDNA, with a specificity of over 99% and a median sensitivity of 43% in stage I tumors, 73% in stage II, and 78% in stage III [35]. Krug et al. [60] collected tumor tissues and blood samples from 84 patients who participated in the TIGER-X test (NCT01526928) and examined ctDNA and ctRNA using the exoNA [detection of RNA (exoRNA) in extracellular vesicle exosomes combined with ctDNA based on NGS] method and the BEAMing (ddPCR) method. The results showed that the exoNA assay had sensitivity of 98% for detecting EGFR mutations and 90% for EGFR T790M mutations, while the sensitivity of the BEAMing method was 82% for EGFR mutations and 84% for EGFR T790M mutations, indicating the advantage of multidimensional combined detection of blood biomarkers.

### 3.5. The Clinical Limitations of Liquid Biopsy Technology

At present, there are several technical limitations in liquid biopsy. Firstly, plasma can be used as a substitute biomaterial instead of tissue to detect tumor information, but its detection sensitivity still needs to be optimized. In clinical practice, it is necessary to fully evaluate its false negative rate, and repeated liquid biopsies or tissue repeat tests should be obtained from patients with negative plasma tests. Secondly, clinical practice of ctDNA analysis lacks a standardized protocol for preanalytical sample preparation and purification. The current analytical testing procedures are complex and may lead to ctDNA degradation and cytolysis. The analytical platform for rapid purification from the blood is a difficult problem to be solved in the future. In addition, challenges in detecting CTCs include extreme rarity, fragility, and physical and phenotypic heterogeneity. Finally, the predictive value of single or small sets of mutations in early detection is limited, and joint detection methods, such as the combination of genomics and transcriptomics or proteomics, may be a new technique to improve tumor-specific detection, like the CancerSEEK platform. Although some studies have confirmed the potential of liquid biopsy to change the mode of cancer treatment, a lot of research is still required in the future to fully define its role in tumor diagnosis, monitoring, and prognosis.

## 4. Summary and Future Directions

Accurate detection techniques promote the development of individualized cancer diagnosis and treatment in the era of precision medicine. At the same time, the demand for clinical precision diagnosis and treatment can further drive the improvement and application of precision detection technology. Precision medicine relies on a thorough understanding of tumor biology. New technologies allow us to map tumors, identify key carcinogenic drivers, and formulate treatments that specifically target tumor cells to achieve clinical benefits. In recent years, precision medical detection techniques promoted the emergence and development of new technologies, new targets, and new drugs in the times of precision medicine. This has accelerated research progress and improved the understanding of disease risk mechanisms and predictions of optimal treatment options for multiple diseases.

At present, there are still many challenges in precision detection techniques and platform construction. Firstly, the results obtained from different NGS-based technologies are inconsistent, which may relate to sample preprocessing, NGS platform, and algorithm differences. Secondly, the sensitivity and accuracy of liquid biopsy need to be verified by further evidence, and it cannot yet be used for early screening and diagnosis of tumors [61]. Thirdly, the cost of single-cell sequencing technology, the need for freshly isolated cells, and the high level of laboratory requirements limit its extensive clinical application. In addition, studies have shown that therapies targeting the same gene mutation have different effects in different tumor types, which may be related to differences in signal transduction and intertumor heterogeneity [62]. The development of precision detection techniques contributes to explore its internal mechanisms and more accurately predict individual patient responses to treatment and achieve individualized precision medical treatment.

In the future, the role of precision detection and combined detection in epigenetics, rare gene detection, individualized targeted therapy, and multigene targeted drug combination therapy should be extensively explored [63]. The accumulation of large samples and big data and the development of cloud computing and artificial intelligence are expected to accelerate scientific research progress, to promote the development of medical science and technology, and to expand the health industry. Precision detection techniques make it possible to understand the molecular characteristics of each patient and the information provided by mutations to develop personalized diagnostic or treatment programs. Also, precision detection techniques contribute to monitoring disease development more closely, evaluating therapeutic effects more scientifically, and fully comprehending the requirements of precision medicine.
